# Pathway Anchored Multimodal Clustering Reveals Circuit Level Signatures in Parkinsons Disease

**DOI:** 10.64898/2025.12.15.694278

**Published:** 2025-12-18

**Authors:** Ashwin Vinod, Aditya Sai Ellendula, Shubham Bhardwaj, Aparna Dev, Aaron Dominic, Chandrajit Bajaj

**Affiliations:** 1 Department of Computer Science, The University of Texas at Austin, Austin, TX, USA; 2 Department of Computer Science, Amrita Vishwa Vidyapeetham, Coimbatore, India

## Abstract

Parkinson’s disease is increasingly understood as a disorder of distributed brain circuits, yet most imaging analyses do not explicitly respect pathway structure. We introduce a pathway-anchored, multimodal clustering framework based on Scalable Robust Variational Compositional Co-clustering (SRVCC) that integrates structural MRI, free-water–corrected diffusion MRI, and DAT-SPECT in anatomically defined circuits. For each pathway, we derive a simple Multimodal Pathway Integrity Score (MPIS) that aggregates z-normalised volume, microstructural, and dopaminergic measures into an interpretable summary of imaging integrity. In the PPMI cohort, SRVCC identifies stable imaging-derived patient clusters and feature modules under explicit model selection and bootstrap/stability checks, with covariate-adjusted analyzes controlling for age, sex, education, and medication. MPIS shows coherent but modest structure–function associations: lower nigrostriatal and frontostriatal integrity relates to higher motor burden (UPDRS-III), while reduced sensory/visuospatial and limbic integrity is linked to lower global cognition (MoCA); microvascular markers robustly stratify imaging profiles but display minimal cross-sectional coupling to these global scales. Feature-level reports highlight dominant region–by–modality contributors (e.g., striatal DAT-SBR, thalamic and cerebellar morphology, white-matter hyperintensity metrics), providing a transparent bridge from multimodal data to circuit-level signatures. This pathway-aware representation offers a principled, reproducible way to summarise multimodal imaging in PD and may support future work on circuit-informed stratification, prognosis, and targeted outcome measures.

## 1 Introduction

Parkinson’s disease (PD) shows substantial heterogeneity in symptoms, progression, and treatment response across patients[17]. In routine care, severity is typically summarized with bedside scales such as the Movement Disorder Society Unified Parkinson’s Disease Rating Scale Part III (MDS-UPDRS III) for motor impairment, the Montreal Cognitive Assessment (MoCA) for global cognition, and the Questionnaire for Impulsive-Compulsive Disorders in Parkinson’s Disease (QUIP) for behavioral symptoms[18, 32, 53]. These instruments standardize symptom scoring but mainly capture functional consequences rather than underlying brain pathology. Neuroimaging provides complementary biological information: dopamine transporter single-photon emission computed tomography (DaT-SPECT) indexes striatal dopaminergic denervation[31, 16], structural MRI captures macroscopic atrophy[22], and diffusion tensor imaging (DTI) and free-water DTI probe white-matter microstructure[36, 42]. Yet many studies evaluate modalities in isolation or prioritize global whole-brain signatures, leaving open whether *multimodal, biology-anchored* integration can expose coherent structure within the PD spectrum.

PD is increasingly understood as a distributed network disorder in which dopaminergic loss in the substantia nigra perturbs basal ganglia–thalamo–cortical loops subserving motor control, executive function, and limbic processing[34]. Heterogeneous involvement of additional systems including frontostriatal and cerebello–thalamo–cortical circuits, cholinergic basal forebrain and pedunculopontine pathways, sensory/visuospatial networks, and microvascular burden contributes to variability in gait, cognition, and behavioral symptoms[45, 39]. Analyses that treat regions as exchangeable features risk groupings driven by coincident covariation rather than mechanism-linked circuitry; a pathway-centric strategy instead respects known anatomical coupling and allows results to be interpreted directly in biological terms, for example as “predominantly nigrostriatal” versus “frontostriatal–executive” involvement.

Over the past decade, multivariate and multimodal frameworks have been developed to fuse imaging modalities and relate them to clinical or genetic variation. Classical approaches such as canonical correlation analysis (CCA)[13] and joint, parallel, or linked independent component analysis (joint/parallel ICA, linked ICA)[49, 19, 30] decompose multimodal data into shared latent components, and multimodal classifiers that combine DaT-SPECT, DTI, and volumetry improve diagnostic or prognostic performance over single-modality markers in PD and atypical parkinsonian syndromes[27, 2]. Large consortia such as ENIGMA-PD have mapped stage-dependent white-matter alterations across Hoehn–Yahr stages using harmonized DTI[39]. More recently, large-scale integration efforts combine longitudinal neuroimaging, CSF, and multi-omics to derive “pace” subtypes and candidate repurposable drugs[48], link gene variants to multimodal brain changes and symptom trajectories via imaging–genomics models[1], and release standardized imaging-derived phenotypes from PPMI to facilitate joint ICA/CCA-style analyses[3]. Other fusion pipelines integrate MRI with genetics[55], diffusion and dopaminergic imaging with clinical scales[54], or neuroimaging with gut microbiome profiles[15] to improve diagnosis or progression prediction. However, these whole-brain or imaging–omics fusion methods typically operate in a latent space whose spatial loadings can be anatomically diffuse and do not explicitly enforce pathway-wise structure or yield patient clusters and feature modules that can be read directly as circuit-level “signatures.”

Unsupervised PD subtyping studies using structural or diffusion features have begun to reveal imaging-defined subgroups with differing motor and cognitive trajectories[23, 58], and supervised radiomics models further distinguish tremor-dominant versus postural-instability/gait-difficulty phenotypes[38]. Yet most existing work either (i) focuses on a single modality, (ii) learns global latent patterns without explicit anatomical binning, or (iii) does not systematically quantify how robust multimodal composites are to modeling choices such as modality weighting, intracranial volume (ICV) normalization, or sign conventions. This motivates an approach that integrates multiple imaging modalities, aggregates features within *predefined neurobiological pathways*, and explicitly assesses robustness and clinical relevance of the resulting circuit-level indices.

Taken together, these observations argue for a representation that preserves modality complementarity and anatomical specificity. In this work, we operationalize a *pathway-anchored stratification* strategy by summarizing multimodal features within each circuit using a *Multimodal Pathway Integrity Score* (MPIS) and then examining (a) pathway-wise separations across patients and (b) structure–function links between MPIS and clinical measures. Instead of assigning opaque, geometry-driven cluster labels, this framework is designed to support statements such as “patients in this subtype show disproportionately low nigrostriatal integrity relative to limbic and sensory pathways.”

We aggregate harmonized structural MRI volumetry, diffusion-derived fractional anisotropy (FA), mean diffusivity (MD) and free water, and DaT-SPECT specific binding ratios within six predefined PD-relevant circuits namely nigrostriatal motor, frontostriatal–executive, limbic/mesolimbic, cerebello–thalamo–cortical, microvascular-burden, and sensory–visuospatial–attention to compute per-subject MPIS for each pathway. We then apply a Scaled Robust Variational Co-Clustering (SRVCC) [52] model to the standardized subject×feature matrix, derive pathway-specific MPIS composites, and examine their separations across imaging-driven patient clusters, as well as their associations with MDS-UPDRS III, MoCA, and QUIP under Benjamini–Hochberg false discovery rate control. In addition, we evaluate MPIS variants that differ in modality weighting and ICV normalization, and quantify SRVCC cluster stability across random initializations and bootstrap resamples.

Our contributions are threefold. First, we present a pathway-stratified SRVCC framework that respects neurobiological circuit organization while enabling data-driven discovery of imaging-defined patient strata and feature modules. Second, we demonstrate that pathway-specific MPIS composites exhibit coherent structure–function relationships aligned with known PD pathophysiology: nigrostriatal and frontostriatal integrity track motor severity, limbic and microvascular pathways relate to behavioral and gait burden, and sensory–visuospatial integrity contributes to cognitive variation. Third, we provide a transparent reporting template linking imaging clusters to specific region–modality biomarkers, pathway-level MPIS profiles, and clinical phenotypes, facilitating replication and mechanistic interpretation.

The remainder of the paper details the cohort, imaging preprocessing, and feature-extraction pipeline (Methods), defines MPIS and the co-clustering and validation procedures, and presents pathway-wise results followed by implications for precision imaging–based stratification in Parkinson’s disease.

## 2 Methods

### 2.1 Participants and Dataset

Data for this study were sourced from the Parkinson’s Progression Markers Initiative (PPMI)[28], a large-scale, open-access longitudinal study aimed at identifying biomarkers of Parkinson’s disease (PD) progression. The dataset includes a total of 294 participants, comprising 185 individuals diagnosed with Parkinson’s disease (62.9%), 72 healthy controls (24.5%), and 37 participants (12.6%) classified as Scans Without Evidence of Dopaminergic Deficiency (SWEDD). Individuals in the PD cohort exhibited two or more cardinal motor signs (rest tremor, bradykinesia, rigidity) along with an abnormal dopaminergic deficit on DaTSCAN SPECT imaging. Healthy controls are individuals without a diagnosis of any neurological disorder, no first-degree family history of PD, normal DaTSCAN results, and no clinical signs of parkinsonism. Participants classified as SWEDD exhibit clinical features resembling parkinsonism but demonstrate normal presynaptic dopaminergic function on DaTSCAN SPECT[7]. All included patients had T1-weighted MRI, diffusion MRI, and DaTSCAN SPECT available for analysis. DaTSCAN SPECT data were used for pathway-specific analyses. Summary demographics (age, sex, education, disease duration, and medication status), along with baseline clinical scores, are reported in Table 1.

#### Clinical Assessments

Standardized clinical evaluations included the Unified Parkinson’s Disease Rating Scale (MDS-UPDRS), the Montreal Cognitive Assessment (MoCA), and the Questionnaire for Impulsive-Compulsive Disorders in Parkinson’s Disease (QUIP). MDS-UPDRS Part III (Motor Examination) is a standardized clinical test to quantify the severity and progression of *motor* features of PD, such as bradykinesia, rigidity, tremor, posture, and gait through structured clinician-rated assessments, with a higher score indicating greater motor impairment[37]. MDS-UPDRS Parts I and II are questionnaires assessing non-motor and motor experiences of daily living, respectively, while Part IV evaluates treatment-related motor complications such as dyskinesias and motor fluctuations. MoCA is a brief 30-point screening tool designed to detect mild cognitive impairment in multiple domains, including attention, executive function, memory, language, visuospatial ability, and orientation[32]. It is widely employed in PD cohorts owing to its high sensitivity in detecting early and subtle cognitive impairments. The Questionnaire for Impulsive-Compulsive Disorders in Parkinson’s Disease (QUIP) is a validated self-administered screening instrument designed to assess impulsive and compulsive behaviors commonly associated with dopaminergic therapy, including pathological gambling, hypersexuality, compulsive buying, binge eating, hobbyism, and behaviors related to medication overuse[53]. It serves as an important clinical measure for identifying behavioral dysregulation and its potential association with dopaminergic treatment. In the feature matrix used for co-clustering, we retained the total numeric score for Part III of the MDS-UPDRS, the MoCA total score, and the QUIP summary score; together these variables define the clinical-only feature view V1 (Table 2).

#### Imaging Modalities

PPMI provides three primary neuroimaging modalities: diffusion tensor imaging (DTI), T1-weighted magnetic resonance imaging (MRI), and DaTSCAN single photon emission computed tomography (SPECT). DTI provides insights into white-matter microstructure and connectivity by quantifying both the directionality (anisotropy) and magnitude of water diffusion within tissue[5]. Voxel-wise maps of fractional anisotropy (FA) and mean diffusivity (MD), derived from DTI, serve as indices of microstructural integrity, with higher FA values reflecting greater directional coherence of diffusion and thus preserved axonal organization. T1-weighted MRI provides high-resolution structural information, enabling the assessment of brain morphology and regional atrophy associated with PD, particularly in regions such as the basal ganglia, thalamus, and frontal cortex[51]. DaTSCAN SPECT is employed as a presynaptic dopaminergic biomarker, leveraging the radiotracer Ioflupane (^123^I, [^123^I]FP-CIT) to visualise dopamine transporter (DAT) density within the striatum; reduced tracer binding reflects the loss of nigrostriatal dopaminergic neurons, enabling differentiation of neurodegenerative parkinsonian syndromes at early stages[6]. These modalities yield regional T1 volumes, diffusion-derived FA/MD (and related microstructural metrics), and striatal DaTSCAN specific binding ratios that are subsequently assembled into multimodal feature sets. For downstream analyses, we define four nested feature views (V1–V4) that progressively add imaging information on top of the clinical scores—from clinical-only (V1) to clinical + T1/DaTSCAN + diffusion/FWE-DTI (V4); the exact composition of each view is summarised in Table 2. Acquisition parameters and modality-specific quality-control procedures, including motion/outlier handling and visual plus quantitative checks of DaTSCAN-to-T1 coregistration, are detailed in Section 2.2.

### 2.2 Imaging Preprocessing and Quality Control

**Figure 2:**
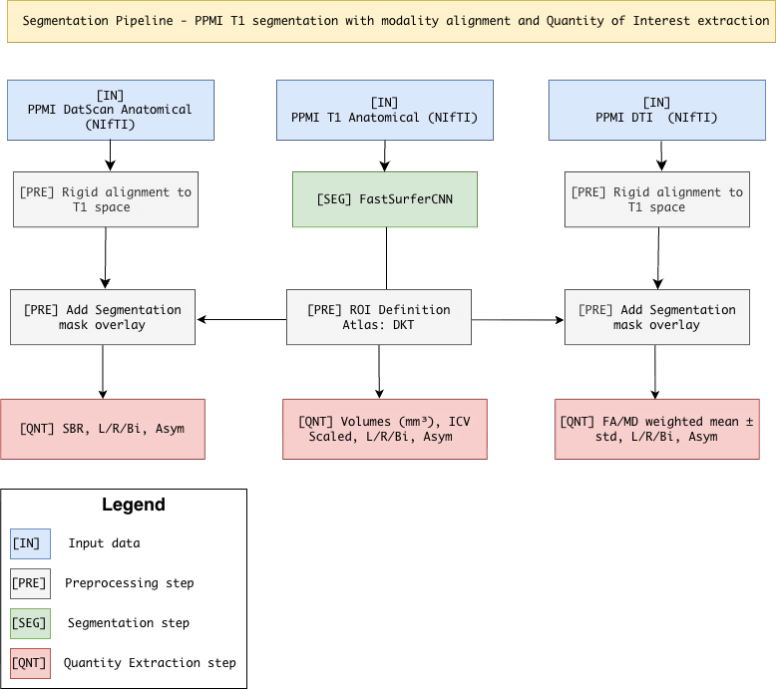
Multimodal segmentation and quantification pipeline.* T1 MRI volumes are segmented with FastSurferCNN to generate DKT-atlas ROIs in subject-native space. These ROIs guide rigid alignment of DaTSCAN SPECT and DTI (FA/MD) images, after which the segmentation mask is overlaid for region-wise quantification: T1 → volumes, DTI → FA/MD weighted mean +− SD, and DaTSCAN → mean uptake and SBR = (ROI/ref). All metrics are computed per hemisphere and bilaterally, with asymmetry indices (Asym = (RL)/(R+L)).

**Figure 3:**
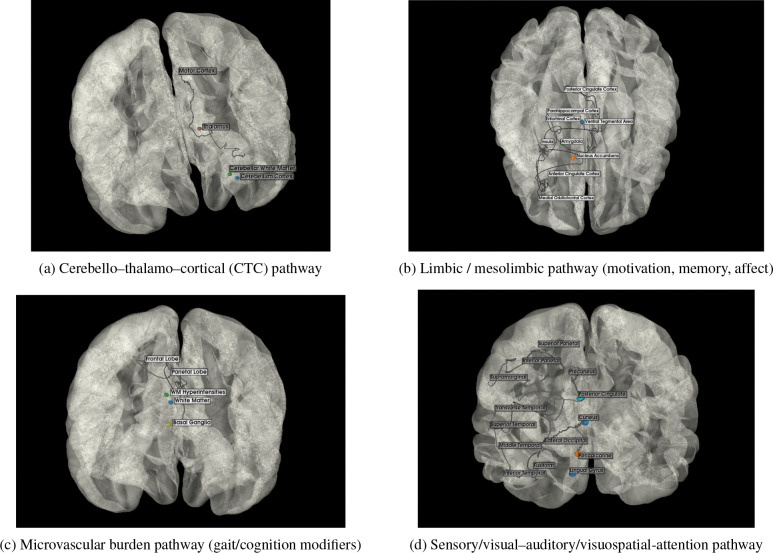
Pathway schematics for (A) cerebello–thalamo–cortical, (B) limbic/mesolimbic, (C) microvascular-burden, and (D) sensory/visual–auditory/visuospatial-attention pathways.

**Figure 4:**
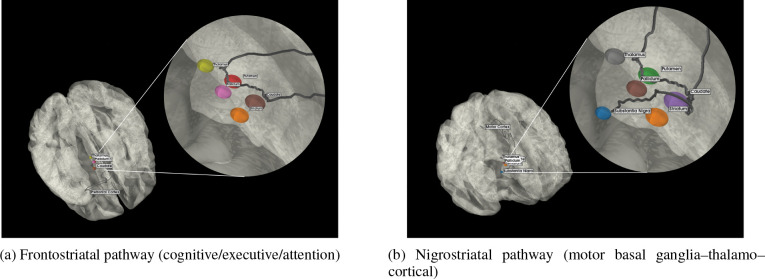
Pathway schematics of (A) frontostriatal (cognitive/executive/attention) and (B) nigrostriatal (motor basal ganglia–thalamo–cortical).

We convert raw multimodal imaging into pathway-aware features through a lean, reproducible pipeline (Fig. 2). First, T1-weighted MRI is segmented to define a subject-specific anatomical reference space; all other modalities are rigidly aligned to this space after skull stripping. Within this reference, we extract ROI-wise structural volumes, diffusion metrics (DTI-FA and DTI-MD), and DAT-SPECT specific binding ratios (SBR). Region-level features are then aggregated using predefined circuit masks; in the next section we formalize this aggregation as the *Multimodal Pathway Integrity Score* (MPIS) and describe clustering and evaluation in pathway space. See the pathway masks in Fig. 3 (CTC, limbic, microvascular, sensory) and Fig. 4 (frontostriatal, nigrostriatal).

We developed a modular pipeline to extract anatomical and microstructural imaging features from multimodal neuroimaging data in Parkinson’s disease (PD), enabling downstream discovery of validated and novel biomarker patterns. The core premise of this pipeline is to define a common anatomical reference frame via whole-brain segmentation, into which diverse imaging modalities can be spatially aligned. This permits region-wise aggregation of biological features across structural MRI, diffusion tensor imaging (DTI), and DaTscan SPECT. Scanner manufacturer, sequence type, key acquisition parameters (field strength, voxel size, and repetition/echo times), and modality-wise inclusion counts before and after quality control are summarized in

#### Anatomical Segmentation and Reference Space

T1-weighted MRI scans were segmented using FastSurferCNN, producing cortical and subcortical labelmaps consistent with the Desikan-Killiany-Tourville (DKT) atlas [20]. Surface reconstruction was not performed in this iteration, so cortical thickness estimates were not included. Cerebellar and hypothalamic substructures were also excluded in the initial segmentation to simplify early-stage harmonization. These labelmaps define the anatomical regions for subsequent feature extraction. All modalities were aligned to this segmentation-defined T1-native space. All T1 volumes and corresponding segmentations were visually inspected in three orthogonal planes to confirm adequate gray–white contrast, absence of major motion or wrap-around artefacts, and correct delineation of major subcortical nuclei; scans with gross artefacts or segmentation failure were excluded from further analysis.

#### Cross-modal Image Registration

Each non-T1 modality was rigidly aligned to the structural T1 image using mutual information–based registration with a multiscale optimization schedule. All modalities were resampled to the T1-native grid using linear interpolation for continuous-valued images. Discrete anatomical labels were retained in their native resolution and not interpolated, ensuring topological consistency for region-based analysis. All registration was performed after skull stripping, and alignment was visually verified through anatomical overlays. For diffusion and DaTSCAN data, registration quality was assessed by overlaying FA/MD maps and DaT uptake maps on the T1 image and examining alignment with segmented cortical and subcortical ROIs. Given the comparatively low spatial resolution and non-anatomical contrast of DaTSCAN SPECT, we additionally required that striatal “hot spots” (high-uptake regions) fall within the union of the caudate and putamen masks in all three planes. For each subject, we computed the center-of-mass of DaT uptake within a broad striatal mask and the ratio of mean uptake inside versus outside the striatal ROIs; scans with a center-of-mass outside the anatomical striatum or with an inside/outside ratio below a prespecified threshold were re-registered, and if satisfactory alignment could not be achieved, the SPECT data for that subject were excluded.

#### Feature Extraction per Modality

We extracted voxelwise scalar maps from each aligned modality and generated region-wise summary measures within anatomical parcels.

- **T1 MRI:** Tissue volumes for cortical and subcortical regions were computed from labelmaps. Volume changes in frontal cortex, basal ganglia, and thalamus are well-documented correlates of motor and cognitive symptoms in PD. Volumes were initially expressed in native units and subsequently converted to intracranial-volume–normalized measures for sensitivity analyses (see Section 2.3); primary analyses report results using non-normalized volumes, with covariate adjustment for age and sex in downstream models.
- **DTI:** Preprocessed fractional anisotropy (FA) and mean diffusivity (MD) maps were aligned to T1 space. No tensor fitting or microstructural modeling (e.g., AD, RD, free-water) was performed in this stage; such metrics are planned for downstream analysis. We inspected FA and MD maps for evidence of severe motion, signal dropout, or ghosting. For each subject, we computed the mean FA and MD within a white-matter mask and flagged scans whose values deviated by more than three standard deviations from the cohort median; flagged scans were manually reviewed and excluded if artefacts were confirmed. Only DTI datasets passing both visual and quantitative quality control were retained for region-wise FA/MD quantification.
- **DaTscan:** SPECT volumes were registered to T1 space and resampled. Specific binding ratios (SBR) were calculated using striatal uptake relative to occipital cortex as a reference region. These values serve as quantitative estimates of presynaptic dopaminergic integrity. In addition to the registration checks described above, all DaTSCAN images were visually screened for truncation, extreme noise, or reconstruction artefacts prior to quantification. Subjects whose DaTSCAN scans failed either quality or registration criteria were retained in structural and diffusion analyses but excluded from DaT-derived metrics.

For multimodal analyses involving MPIS computation and Scaled Robust Variational Co-Clustering (SRVCC), we restricted the cohort to participants with T1 MRI, DTI (FA and MD), and DaTSCAN SPECT that passed the above quality control procedures in all three modalities. The resulting effective sample size for multimodal analyses, along with modality-wise inclusion and exclusion counts, is summarized in Table 3.

### 2.3 Feature construction and Multimodal Pathway Integrity Score (MPIS)

Quantifying disease-related degradation within distributed brain circuits has historically relied on single-modality scalar biomarkers such as the Parkinson’s Disease–Related Pattern (PDRP) expression score from FDG-PET, which summarizes metabolic network dysfunction at the subject level [29], or on hybrid PET–MRI indices linking dopaminergic denervation to microstructural disorganization in the nigrostriatal pathway [47]. Building upon these precedents, we define the **Multimodal Pathway Integrity Score (MPIS)** as a unified, pathway-specific composite integrating structural, diffusion, and dopaminergic features into a single interpretable index of circuit integrity.

Building on the QC’ed T1, DTI, and DaTSCAN data described in Section 2.2, we construct a unified set of regional imaging features and compress them into pathway-level integrity scores. At the subject level, we assemble a subject×feature matrix whose columns are ROI–modality measurements (T1 volumes, FA/MD from DTI, and striatal SBR from DaTSCAN), together with selected bilateral and asymmetry summaries. For downstream analyses and ablations, these features are organized into four nested views (V1–V4) that progressively add structural, dopaminergic, and diffusion information, as summarized in Table 2. To impose a biologically interpretable circuit structure, we further group ROIs into six PD-relevant pathway bins (nigrostriatal, frontostriatal–executive, cerebello–thalamo–cortical, limbic/mesolimbic, microvascular-burden, and sensory/visuospatial), whose ROI and modality composition is specified in Table 4 and illustrated schematically in Figures 3 and 4.

The following subsections detail (i) how ROI-level features are constructed and binned into pathways, (ii) how MPIS is computed from these components, and (iii) how we assess the robustness of our conclusions to alternative MPIS formulations.

#### ROI-level feature construction and pathway binning

From the preprocessing pipeline described above, we obtain, for each participant, regional features derived from T1-weighted MRI (volumes), DTI (FA and MD), and DaTSCAN SPECT (striatal uptake and specific binding ratios, SBR). Using the DKT-based segmentation in T1-native space, we compute left and right hemisphere values for each ROI and modality and, where appropriate, bilateral means and asymmetry indices (Asym=(R–L)/(R+L)). These regional measurements instantiate the columns of the subject×feature matrix $X\in R^{N\times D}$ introduced above, with each row representing one participant and each column one ROI–modality–hemisphere (or asymmetry) feature. Non-numeric metadata fields and derived “<mono@space>-std</mono@space>” columns are excluded. Features are standardized via a global z-transform (subtracting the cohort mean and dividing by the cohort standard deviation), so that each column of X has zero mean and unit variance before downstream analyses.

To operationalize a “pathway-centric” representation, we then map these ROI-level features into the predefined circuit bins summarized in Table 4. Consistent with the overview above, anatomically related ROIs are grouped into six PD-relevant pathways (nigrostriatal, frontostriatal–executive, cerebello–thalamo–cortical, limbic/mesolimbic, microvascular-burden, and sensory/visuospatial). For each pathway, we specify (i) the set of cortical and subcortical ROIs assigned to the circuit and (ii) the modalities (volume, FA, MD, SBR) available for those ROIs. Figures 3 and 4 provide schematic illustrations of these circuits, while Table 4 makes explicit the mapping from the abstract notion of “pathway-anchored stratification” to the underlying ROI–modality features used in our analyses.

#### Definition of MPIS

For every predefined pathway (see Fig. 4a–b and Fig. 3a–d), all contributing imaging features (fractional anisotropy, mean diffusivity, volumetric measures, and striatal binding ratios) were *z*-scored at the ROI–modality level. We then construct a pathway-specific composite that summarizes, for each subject, the balance of structural and dopaminergic integrity within that circuit. To maintain an interpretable directionality across modalities, FA, volumes, and SBR enter the composite with positive sign (higher values reflect greater integrity), whereas MD enters with a negative sign (higher MD typically reflects microstructural degradation). Volumes are used in their native units for the primary MPIS definition; in Section 2.5 we additionally adjust for age and sex and report sensitivity analyses using intracranial-volume (ICV)–normalized volumes to assess robustness.

As a simple, a priori baseline, we assign equal numeric weights to all modalities within a pathway (i.e., $w_{FA}=w_{MD}=w_{\text{VOL}\;}=w_{\text{SBR}\;}=1$, with the MD term entering with a negative sign). This choice avoids post hoc tuning and keeps the composite directly interpretable as an average standardized deviation across all available features in the circuit. We explicitly examine alternative weighting schemes in robustness analyses, including modality- and pathway-specific weights that emphasize dopaminergic markers in the nigrostriatal system and volumetric measures in cortical pathways.

The resulting composite was then standardized across subjects to yield a dimensionless MPIS: higher values indicate a pathway-specific feature pattern aligned with greater structural/binding integrity, whereas lower values reflect relative decrements (e.g., reduced FA, elevated MD) within that circuit. MPIS is computed from z-scored features per pathway; we evaluate its association with clinical scores and its separation across pathway-wise clusters in downstream analyses.

Formally, for subject i and pathway p,

$${MPIS}_{i}^{\left(p\right)}=𝒵\left(\sum_{j\in {ℱ}_{p}^{FA}}\;FA_{ij}^{\left(p\right)}-\sum_{j\in {ℱ}_{p}^{MD}}\;MD_{ij}^{\left(p\right)}+\sum_{j\in {ℱ}_{p}^{VOL}}\;VOL_{ij}^{\left(p\right)}+\sum_{j\in {ℱ}_{p}^{SBR}}\;SBR_{ij}^{\left(p\right)}\right),$$

where $FA_{ij}^{\left(p\right)}$, $MD_{ij}^{\left(p\right)}$, $VOL_{ij}^{\left(p\right)}$, and $SBR_{ij}^{\left(p\right)}$ denote the z-scored modality values for feature j within pathway p, and ${ℱ}_{p}^{\left(\cdot \right)}$ is the set of indices for each modality in pathway p. The operator 𝒵(⋅) standardizes the composite across subjects so that ${MPIS}_{i}^{\left(p\right)}$ has zero mean and unit variance for pathway p.

#### MPIS robustness and sensitivity analyses

Because the MPIS definition involves modeling choices (equal modality weights, sign conventions, and the use of non-ICV-normalized volumes), we explicitly quantify the robustness of our findings to these choices. We define a family of MPIS variants for each pathway:

- *Equal-weights MPIS (primary):* the definition above, with all modalities equally weighted and MD entering with negative sign.
- *ICV-normalized MPIS:* identical to the primary definition, but with ROI volumes replaced by ICV-normalized volumes (volume / ICV) prior to z-scoring.
- *Pathway-weighted MPIS:* a variant in which modality weights are allowed to differ by pathway (e.g., upweighting SBR in the nigrostriatal pathway and downweighting volumes in microvascular-burden regions) based on established PD pathophysiology.
- *Non-signed MD MPIS:* a control variant in which MD enters with positive sign, so that higher MPIS reflects a mixture of increased FA and increased MD; this variant tests the extent to which our results depend on enforcing a uniform “higher = more intact” direction across modalities.

For each variant, we recompute pathway-wise MPIS values and recover their associations with MDS-UPDRS III, MoCA, and QUIP_SUM, as well as their separation across Scaled Robust Variational Co-Clustering (SRVCC) patient clusters. We report (i) Pearson and Spearman correlations between the primary MPIS and each variant, (ii) concordance in the sign and FDR-corrected significance of MPIS–clinical associations, and (iii) the stability of between-cluster MPIS contrasts (effect sizes and rank order of clusters). These robustness analyses demonstrate that the main conclusions of the paper i.e., the existence of distinguishable pathway-level signatures linked to motor, cognitive, and behavioral burden are not driven by a single arbitrary choice of weighting, normalization, or sign convention.

### 2.4 Scalable Robust Variational Compositional Co-clustering (SRVCC) and cluster validation

We use a variational co-clustering model that jointly learns patient and feature clusters from the full subject × feature matrix. Post hoc, we compute pathway-level MPIS and relate them to clinical measures. All clustering analyses are performed on the multimodal QC subset described in Sections 2.1–2.2, using the z-scored ROI–modality features defined in Section 2.3.

#### Data and preprocessing.

Let $X\in R^{N\times D}$ denote the subject × feature matrix parsed from the CSV after (i) dropping non-numeric fields and any “<mono@space>-std</mono@space>” columns, (ii) removing rows with NaN/inf or all-zero modality values, and (iii) standardizing each retained feature via a global z-transform. For model stability we also apply per-row and per-column normalization inside the training pipeline. When cohort labels are present, mini-batches are sampled with inverse-frequency weights to mitigate cohort imbalance. No clinical variables (e.g., age, sex, diagnosis, medication status) are used to define clusters; these covariates are only incorporated later when relating clusters and MPIS to clinical outcomes (Section 2.5).

#### SRVCC co-clustering.

We train two SRVCC modules [52]: a *row* model on X (patients) and a *column* model on $X^{⊤}$ (features). For the row side, the encoder produces $q_{\phi }\left(z_{i}|x_{i}\right)=𝒩\left(\mu_{i},\|\sigma_{i}^{2}\right)$ and the decoder reconstructs ${\overset{ˆ}{x}}_{i}=f_{\theta }\left(z_{i}\right)$. The latent prior is a Gaussian mixture

$$p\left(z\right)=\sum_{k=1}^{K_{r}}\pi_{k}𝒩\left(z|\mu_{k},diag\;\sigma_{k}^{2}\right),$$

with learnable mixture log-weights, means, and (diagonal) covariances. Responsibilities for a latent sample $z_{i}$ are

$$\gamma_{ik}\propto \pi_{k}𝒩\left(z_{i}|\mu_{k},diag\sigma_{k}^{2}\right),\;\sum_{k}\gamma_{ik}=1.$$

The row loss is a SRVCC objective,

$${ℒ}_{\text{row}\;}=\sum_{i=1}^{N}\underset{\text{MSE}\;\text{recon}\;}{\underbrace{{\left\|x_{i}-{\overset{ˆ}{x}}_{i}\right\|}_{2}^{2}}}+\beta \;KL\left(q_{\phi }\left(z_{i}|x_{i}\right)\|p(z)\right),$$

and analogously for the column side with $K_{c}$ components. We couple the two factorizations with a mutual-information term. Let $\Gamma_{r}\in R^{N\times K_{r}}$ and $\Gamma_{c}\in R^{D\times K_{c}}$ be the soft assignment matrices. Define a normalized cross-tabulation

$$T_{org}=\frac{\Gamma_{r}^{⊤}\Gamma_{c}}{1^{⊤}\left(\Gamma_{r}^{⊤}\Gamma_{c}\right)1}\in R^{K_{r}\times K_{c}},$$

and its “reduced” counterpart $T_{\text{red}}$ formed by replacing rows of $\Gamma_{r}$ and $\Gamma_{c}$ with one-hot hard assignments. With $MI(T)=\sum_{ab}T_{ab}log\left(T_{ab}/\left(T_{a}\cdot T_{\cdot }b\right)\right)$,

$${ℒ}_{MI}=\lambda_{mi}\;log\left(1+\left|1-MI\left(T_{red}\right)/MI\left(T_{\text{org}}\right)\right|\right).$$

The total objective is

$$ℒ={ℒ}_{row}+{ℒ}_{col}+{ℒ}_{MI}.$$

Training proceeds as: (1) pretrain each VAE (no mixture prior) for several epochs; (2) initialize GMM parameters by k-means on encoder means; (3) jointly optimize with Adam using a KL warm-up schedule (β↑1). We set default $K_{r}=K_{c}=5$ and automatically cap by the available samples/features. Final hard row/column clusters are <mono@space>arg max</mono@space> of the responsibilities. Note that SRVCC operates directly on the standardized ROI–modality feature matrix X; MPIS is computed *after* clustering and is used solely for pathway-level interpretation and clinical association analyses.

#### Choice of number of clusters.

To avoid an arbitrary choice of patient and feature cluster numbers, we perform an explicit model selection over $\left(K_{r},K_{c}\right)\in \{3,\ldots ,7{\}}^{2}$. For each candidate pair, we train SRVCC with five random initializations and compute: (i) the final variational objective ℒ (lower is better), (ii) held-out reconstruction error on a 20% validation set, and (iii) the mutual-information ratio $MI\left(T_{\text{org}}\right)/MI\left(T_{\text{red}}\right)$, which captures how well the soft co-clustering structure is preserved after hard assignment. We select the ($K_{r},K_{c}$) configuration that achieves a favorable trade-off between goodness-of-fit (low reconstruction error, low ℒ) and parsimony (no unnecessary increase in $K_{r}$, $K_{c}$). The corresponding model-selection summary is reported in the Results (cluster-quality metrics versus $\left(K_{r},K_{c}\right)$).

#### Stability and reproducibility of SRVCC clusters.

To assess the robustness of patient and feature clusters, we quantify stability across random initializations and across bootstrap resamples of the cohort. First, for the selected ($K_{r},K_{c}$), we retrain SRVCC 10 times with different random seeds and compute pairwise adjusted Rand index (ARI) and normalized mutual information (NMI) between the resulting hard patient-cluster assignments; high median ARI/NMI indicates that the row clusters are not driven by initialization. Second, we perform a nonparametric bootstrap (100 resamples of 80% of subjects drawn with replacement), fit SRVCC on each resample, and compare the resulting clusters to the full-sample solution using ARI/NMI. We report the distribution of these stability metrics in the Results, demonstrating that the learned patient strata and feature groupings are reproducible under resampling.

These empirical stability analyses are complemented by prior large-scale evaluations of SRVCC, which demonstrate consistently high clustering accuracy and NMI across diverse datasets and experimental settings, with low variance across random initializations (Supplementary Tables 29–30). Together, these results support the robustness of SRVCC to initialization, sampling variability, and data modality.

#### Cluster composition and covariates.

For interpretability, we summarize the composition of each patient cluster by diagnosis (PD, healthy control, SWEDD), sex, medication status, and scanner field strength. Cluster-level cross-tabulations and continuous summaries (age, disease duration, clinical scores) are reported in Table 5. When relating cluster membership to clinical outcomes, we use regression models that adjust for age, sex, education, and medication status, and in sensitivity analyses we additionally include scanner field strength as a covariate (Section 2.5). This separation by using imaging features alone to learn clusters, then adjusting for covariates when testing clinical associations addresses concerns about confounding while keeping the clustering step biologically agnostic.

### 2.5 Statistical analysis and pathway-aware evaluation

#### Primary outcomes and covariates

Our primary clinical outcomes were the MDS-UPDRS Part III motor score (MDS-UPDRS III), the Montreal Cognitive Assessment (MoCA), and the total score on the Questionnaire for Impulsive-Compulsive Disorders in Parkinson’s Disease (QUIP_SUM). These measures were selected to capture motor severity, global cognition, and behavioral dysregulation, respectively.

Unless otherwise specified, all regression-based analyses adjusted for age, sex, years of education, dopaminergic medication status (on/off at imaging), and scanner field strength (1.5T vs. 3T). In PD-only models, we additionally included disease duration as a covariate. Continuous variables were inspected for outliers and approximate normality; where appropriate, we used nonparametric tests or robust effect-size measures. Multiple comparisons across pathways and outcomes were controlled using the Benjamini–Hochberg false discovery rate (FDR) procedure at q=0.05. For all key statistics we report point estimates together with 95% confidence intervals (CIs).

#### MPIS–clinical associations (pathway-aware evaluation)

For each pathway we (i) test rank correlations between ${MPIS}_{ig}$ and available clinical measures (UPDRS-III, MoCA, QUIP_SUM) using Spearman ρ with Benjamini–Hochberg FDR correction across outcomes; (ii) assess separation of ${MPIS}_{ig}$ across learned row clusters via Kruskal–Wallis H with $\eta^{2}$ effect size; and (iii) report Cliff’s δ for a pre-specified cluster contrast (0 vs. 3). Heuristic expectation checks flag notable deviations (e.g., non-negative ρ for pathways expected to anticorrelate with UPDRS-III). These statistics, together with the feature-level separation metrics and cohort composition summaries, support mechanism-aligned interpretation of the learned strata.

To quantify uncertainty in these associations, we obtained 95% CIs for Spearman ρ and Cliff’s δ via nonparametric bootstrap (1,000 resamples of subjects with replacement). For Kruskal–Wallis tests we report both the H statistic and an $\eta^{2}$ effect size, along with FDR-adjusted p-values across pathways and outcomes.

In addition to rank correlations, we fit covariate-adjusted linear regression models with each clinical score as the dependent variable and pathway-specific MPIS as the predictor of interest, controlling for age, sex, education, dopamine-ergic medication status, and scanner field strength (and disease duration in PD-only analyses). Regression coefficients for MPIS, together with 95% CIs and FDR-corrected p-values, are reported in the Results. These models assess whether pathway integrity adds explanatory value for motor, cognitive, or behavioral outcomes beyond demographic and treatment-related covariates.

All MPIS–clinical analyses used the pathway scores defined in Section 2.3 and were restricted to subjects with multimodal imaging that passed quality control (Sections 2.2–2.4).

### Cluster-wise comparisons and uncertainty quantification

We next examined how the SRVCC-derived patient clusters differ in terms of clinical outcomes, covariates, and pathway-level MPIS. For continuous variables (e.g., age, disease duration, MDS-UPDRS III, MoCA, QUIP_SUM, and pathway MPIS), we used Kruskal–Wallis tests to evaluate overall differences across clusters, reporting $\eta^{2}$ as an effect size and FDR-adjusted p-values. For categorical variables (e.g., diagnosis group [PD/HC/SWEDD], sex, medication status, scanner field strength), we used $\chi^{2}$ tests of independence or Fisher’s exact tests when counts were sparse, reporting Cramér’s V as an effect size.

To characterize specific contrasts of interest (for example, between a cluster enriched for dopaminergic denervation and a cluster with relatively preserved integrity), we computed pairwise Cliff’s δ between clusters for key continuous outcomes and pathway MPIS values. As above, 95% CIs for Cliff’s δ were obtained via nonparametric bootstrap. This combination of omnibus nonparametric tests and robust effect sizes helps disentangle statistically significant but clinically small differences from more substantial, pathway-aligned separations.

Finally, to assess whether cluster membership explained additional variance in clinical scores beyond covariates, we fit linear regression models with clinical scores as outcomes, including cluster indicators as predictors and adjusting for age, sex, education, medication status, scanner field strength, and (for PD-only models) disease duration. We report global F-tests (or likelihood-ratio tests for nested models), partial $R^{2}$ for the cluster terms, and FDR-corrected p-values.

#### MPIS sensitivity analyses

Because the Multimodal Pathway Integrity Score (MPIS) relies on modeling choices (e.g., equal modality weights and the absence or presence of intracranial volume normalization), we performed sensitivity analyses to evaluate the robustness of our findings. Specifically, we constructed three MPIS specifications: (i) the primary definition from Section 2.3 (no ICV normalization, equal modality weights across FA, MD, volume, and SBR), (ii) an ICV-normalized variant in which regional volumes were adjusted by intracranial volume before z-scoring, and (iii) a modality-reweighted variant in which SBR received higher weight in the nigrostriatal pathway and volume contributed more strongly in microvascular pathways, guided by prior imaging literature.

For each MPIS specification we recomputed pathway scores and repeated the MPIS–clinical association analyses described above. We quantified robustness by (a) the proportion of MPIS–clinical associations that remained directionally consistent and FDR-significant across specifications, and (b) the rank correlation between effect sizes (Spearman ρ and regression coefficients) across MPIS variants. These sensitivity results are summarized in the Results, and demonstrate that the main pathway-level conclusions do not hinge on a single arbitrary choice of MPIS weighting or ICV normalization.

#### Data availability

The imaging and clinical data analysed in this study were obtained from the Parkinson’s Progression Markers Initiative (PPMI). Because these are third-party human-subject data under a data-use agreement, the raw data cannot be redistributed by the authors; qualified researchers can apply for access via PPMI.

#### Code availability

All custom code used to preprocess imaging data, compute the Multimodal Pathway Integrity Score (MPIS), and implement the SRVCC-based co-clustering model is provided in a publicly accessible repository (available here). The repository includes configuration files and scripts to reproduce the main analyses and figures from the manuscript, together with minimal documentation.

## 3 Results

### 3.1 Global imaging-driven patient clusters

We first examined the global structure of multimodal imaging variation by applying the SRVCC compositional co-clustering framework (Section 2.4) to the full feature view V 4 (clinical + T1 + DaTSCAN + FWE-DTI). This yielded a block-structured patient–by–feature matrix with K imaging-driven patient clusters and L feature modules spanning dopaminergic signal (DaT-SBR), diffusion microstructure (FA/MD/FW), and regional morphology. The resulting checkerboard pattern shows that patients within the same cluster share coherent multimodal profiles, while feature modules map onto neuroanatomically and modality-consistent systems that recapitulate the pathway definitions in Tables 6 and 7.

To avoid an arbitrary choice of the number of patient and feature clusters, we carried out the explicit SRVCC model selection described in Section 2.4, scanning $\left(K_{r},K_{c}\right)\in \{3,\ldots ,7{\}}^{2}$. For each candidate pair we computed (i) the final variational objective ℒ, (ii) held-out reconstruction error on a 20% validation split, and (iii) the mutual-information ratio $MI\left(T_{\text{org}}\right)/MI\left(T_{\text{red}}\right)$, which quantifies how much of the soft co-clustering structure is preserved after hard assignment. As summarized in Table 8a, both reconstruction error and ℒ decreased steeply up to a configuration ($K_{r}^{⋆},K_{c}^{⋆}$) and then showed diminishing returns for larger models, while the mutual-information ratio plateaued. We therefore adopted ($K_{r}^{⋆},K_{c}^{⋆}$) as the working configuration for all subsequent analyses.

We then quantified the stability and reproducibility of this selected SRVCC solution. For ($K_{r}^{⋆},K_{c}^{⋆}$), we retrained the model 10 times with different random seeds and computed pairwise adjusted Rand index (ARI) and normalized mutual information (NMI) between the resulting hard patient-cluster assignments. Median ARI and NMI were high with narrow interquartile ranges (Table 8b), indicating that the row clusters are not driven by initialization. In a complementary nonparametric bootstrap analysis (100 resamples of 80% of subjects drawn with replacement), we refit SRVCC on each resample and compared the resulting clusters to the full-cohort solution using ARI/NMI; the resulting distributions, also summarized in Table 8b, again showed high concordance, demonstrating that the learned patient strata and feature modules are robust to sampling variability. Repeating SRVCC on alternative feature views (V2 and V3) produced similar coarse-grained cluster structure and high agreement with the V 4 row assignments (pairwise ARI > threshold), suggesting that the global co-clustering is driven by robust cross-modal covariance rather than idiosyncratic features of a single view.

Importantly, these global imaging-derived clusters already exhibit recognizable clinical gradients. Clusters characterized by lower striatal DaT-SBR and less favorable diffusion/morphometric profiles align qualitatively with higher motor burden, whereas clusters with preserved dopaminergic and posterior cortical integrity show relatively better cognitive performance. These trends are quantified more formally in our cluster-wise clinical comparisons and covariate-adjusted regression models below (Sections 3.2 and 2.5).

Because clustering used imaging features only and clinical variables were linked post hoc, any observed imaging–phenotype associations reflect emergent structure–function relationships rather than circularity.

In the remainder of the Results, we leverage this global, imaging-driven cluster solution in two complementary ways: (i) by summarizing how standard covariates and diagnoses distribute across clusters (Section 3.2), and (ii) by probing, for each predefined pathway, how an interpretable Multimodal Pathway Integrity Score (MPIS) and key features relate to these global clusters and to clinical scales.

### 3.2 Cluster composition and covariates

To assess potential confounding and to clarify the clinical meaning of the imaging-driven clusters, we first examined their composition with respect to diagnosis and standard demographic/technical covariates. Table 5 summarizes, for each global cluster, the number of participants and the distribution of baseline diagnosis (PD, SWEDD, control), age, sex, years of education, disease duration (for PD/SWEDD), levodopa-equivalent daily dose, and scanner field strength, as well as the marginal distributions of the primary clinical scales.

By construction, the SRVCC clustering algorithm did not use any clinical or covariate information; clusters are defined solely in the multimodal imaging space. Consistent with this design, the covariate summaries in Table 5 show no extreme demographic or acquisition imbalances that would trivially explain the observed imaging structure (for example, no cluster is composed exclusively of a single diagnostic group or a single scanner field strength). Instead, PD and SWEDD participants are represented across multiple clusters, and age, sex, and education show overlapping ranges, suggesting that the global clusters primarily capture latent imaging phenotypes rather than obvious sampling artefacts.

These descriptive patterns motivate the covariate-adjusted analyses reported in Section 2.5, where we quantify how much additional variance in motor severity, global cognition, and impulsivity/compulsivity (QUIP_SUM) is explained by cluster membership after controlling for age, sex, education, medication status, scanner field strength, and disease duration. Together, Sections 3.2 and 2.5 address concerns that the discovered imaging clusters might simply recapitulate demographic or acquisition differences, and provide a principled baseline for interpreting the pathway-specific MPIS results that follow.

### 3.3 Pathway-level MPIS and clinical associations

We quantify pathway-specific imaging heterogeneity and its relationships to clinical measures using multimodal features spanning dopaminergic signal (DaT-SBR), diffusion microstructure (FA/MD), and regional morphology (with optional ICV scaling), we derived a Multimodal Pathway Integrity Score (MPIS) and data-driven clusters for each circuit of interest.

For each predefined pathway mask (nigrostriatal motor, frontostriatal executive, sensory/visual–visuospatial, limbic/mesolimbic, microvascular burden, and cerebello–thalamo–cortical balance), we recomputed MPIS from the subset of features belonging to that circuit and linked MPIS to motor severity (MDS-UPDRS III), global cognition (MoCA), and impulsivity/compulsivity (QUIP_SUM) using Spearman correlations with BH–FDR correction. Table 9 summarizes, for each pathway, (i) the Kruskal–Wallis separation of MPIS across its imaging clusters and (ii) the strongest MPIS–clinical associations that survived or approached FDR control.

For each pathway and clinical scale, Table 9 reports Spearman correlations together with 95% bootstrap confidence intervals and FDR-adjusted q-values, making explicit the uncertainty around the MPIS–clinical associations.

Quantitatively, the largest MPIS–motor effects were observed in the nigrostriatal and frontostriatal pathways (MDS-UPDRS III ρ≈−0.20 and ρ≈−0.19, respectively; both FDR-significant), indicating that lower dopaminergic and microstructural integrity in these circuits aligns with higher motor burden. The strongest MPIS–cognition effects occurred in the sensory/visual–visuospatial and limbic pathways (MoCA ρ≈0.16 and ρ≈0.12, respectively; FDR-significant or trending), consistent with posterior cortical and limbic contributions to global cognitive performance. In contrast, the microvascular pathway showed very strong imaging stratification (large MPIS separation across clusters) but near-zero correlations with MDS-UPDRS III, MoCA, or QUIP_SUM, suggesting that its functional impact may be more apparent for gait- and dysexecutive-specific endpoints than for the global scales used here.

Effect sizes were generally modest, as expected for single-pathway summaries in heterogeneous PD cohorts, but they were directionally coherent, statistically controlled (BH–FDR), and aligned with established circuit-level models of PD pathophysiology. In the following subsections, we illustrate these patterns with representative pathway-specific cluster profiles and MPIS–clinical plots, using the global SRVCC clusters from Section 3.1 as a common reference frame.

We verified that these pathway-level patterns are robust to common modeling choices in MPIS construction. Specifically, we repeated all analyses using (i) ICV-normalized volumes, (ii) a modality-reweighted MPIS that balances variance contributions from DaT-SBR, diffusion, and volumetry, and (iii) a control specification that retains the original sign of mean diffusivity. Across pathways, primary MPIS values were highly correlated with all variants (median Pearson r∼0.96) and MPIS–clinical associations (sign and FDR-adjusted significance pattern) were essentially unchanged (Appendix 5.1), indicating that our conclusions do not hinge on a single arbitrary MPIS definition.

To confirm that these pathway–clinical links are not driven by demographic or acquisition covariates, we also fitted covariate-adjusted linear models with each clinical scale as outcome and MPIS as the primary predictor, controlling for age, sex, education, disease duration, levodopa-equivalent daily dose, and scanner field strength. The resulting $\beta_{\text{MPIS}}$ coefficients and 95% confidence intervals (Appendix 5.2, Table 28) remained directionally consistent with the rank-based correlations in Table 9 and of similar magnitude, indicating that the observed MPIS–clinical associations are robust to these covariates.

### 3.4 Pathway-specific profiles

Table 9 summarizes cross-pathway MPIS separation across pathway-derived clusters and MPIS–clinical associations (BH–FDR). To reduce repetition in the main Results, we highlight two representative pathways here—one motor-anchored (nigrostriatal) and one cognition-anchored (sensory/visuospatial). Full per-pathway reporting for all circuits (Kruskal–Wallis tables, MPIS summary tables, top-feature tables, heatmaps, and ranked standardized-gap plots) is provided in Supplementary Results, using the same figure and table reference labels as in this manuscript.

#### Nigrostriatal Motor (BG–thalamo–cortical) Pathway

The nigrostriatal pathway showed the clearest MPIS–motor coupling (Table 9): lower MPIS was associated with higher motor severity (MDS-UPDRS III: ρ≈−0.201, $q\approx 6.8\times 10^{-4}$), with negligible associations with MoCA and QUIP_SUM. MPIS also separated strongly across pathway clusters ($H_{\text{MPIS}}\approx 167.15,\eta^{2}\approx 0.587$), indicating robust within-pathway stratification. Detailed per-outcome cluster tests and feature-driver analyses are reported in Supplementary Results (see Tables 11–13 and Figs. 8–10).

#### Sensory / Visual / Auditory and Visuospatial-Attention Pathway

The sensory/visuospatial pathway showed the strongest MPIS–cognition association (Table 9): higher MPIS was associated with better global cognition (MoCA: ρ≈0.163, q≈0.0071), with weaker non-significant relationships to MDS-UPDRS III and QUIP_SUM. MPIS separated strongly across pathway clusters ($H_{\text{MPIS}}\approx 170.68,\eta^{2}\approx 0.629$). Full per-outcome cluster tests and feature-driver analyses are reported in Supplementary Results (see Tables 18–20 and Figs. 16–18).

#### Frontostriatal Cognitive (Executive/Attention) Pathway

Frontostriatal MPIS showed a robust negative association with motor severity (MDS-UPDRS III: ρ≈−0.191,q≈0.0014) and strong MPIS separation across clusters ($H_{\text{MPIS}}\approx 121.50,\eta^{2}\approx 0.432$), with weaker global cognitive and QUIP_SUM associations (Table 9). Full figures and tables are provided in Supplementary Results (Figs. 11–14; Tables 14–17).

#### Limbic / Mesolimbic Pathway

Limbic MPIS showed robust imaging stratification ($H_{\text{MPIS}}\approx 165.25,\eta^{2}\approx 0.58$) with a modest positive association with cognition (MoCA: ρ≈0.119, q≈0.045) and weaker motor/behavioral coupling (Table 9). Full figures and tables are provided in Supplementary Results (Fig. 19 and Fig. 20; Tables 21 and 23).

#### Microvascular Burden (Gait/Cognition Modifiers) Pathway

Microvascular MPIS stratified imaging profiles across clusters ($H_{\text{MPIS}}\approx 127.11,\eta^{2}\approx 0.445$) but showed near-zero correlations with MDS-UPDRS III/MoCA/QUIP_SUM (all q≫0.1; Table 9), consistent with a modifier interpretation and motivating more targeted gait/dysexecutive endpoints. Full figures and tables are provided in Supplementary Results (Figs. 22–25; Tables 24–26).

#### Cerebello–thalamo–cortical (Balance) Pathway

CTC/balance results in this run were imaging-only (MPIS–clinical correlations not computed), but clusters separated strongly with dominant contributions from cerebellar morphometry and diffusion metrics. Full figures and the top-feature summary are provided in Supplementary Results (Figs. 26–29; Table 27).

#### Ablation Across Feature Views

We evaluated robustness of the co-clustering and MPIS construction across four feature views (2) ranging from clinical-only to the full multimodal set.

With clinical scores alone (V1), the resulting clusters were coarser, less stable across resamples, and offered limited anatomical specificity: cluster-defining differences primarily reflected overall severity gradients rather than distinct circuit-level profiles. Adding structural volumes and DaT-SBR (V2) sharpened separation, particularly in striatal and limbic systems, and improved alignment with motor burden, but still yielded weaker microstructural differentiation. Incorporating diffusion metrics (V3) produced further gains in within-cluster homogeneity, although uncorrected DTI introduced modest redundancy with T1 volumes. The full multimodal configuration with free-water–corrected diffusion (V4; primary analysis) provided the most stable clusters and clearest pathway-level interpretations, with MPIS patterns and top separating features consistent across bootstrap runs. Together, these ablations indicate that our pathway-specific conclusions are not driven by a single modality, but reflect convergent signal from structure, dopaminergic binding, and tissue microstructure.

#### Pathway–Global Concordance

To assess whether pathway-anchored clusters recapitulate the global patient subtypes, we compared pathway-specific co-clusters with the global SRVCC-derived clusters using normalized mutual information (NMI) and adjusted Rand index (ARI; 10).

Nigrostriatal co-clusters showed the strongest concordance with global subtypes (NMI ≈ 0.72, ARI ≈ 0.68), followed by frontostriatal executive, hippocampal memory, cerebellar motor, and mesolimbic reward pathways, all well above permutation baselines (NMI ≈ 0.08, ARI ≈ 0.02). Hypothalamic/autonomic clusters exhibited lower, borderline-significant concordance, consistent with a more modulatory role. This gradient suggests that global multimodal subtypes are anchored primarily in motor nigrostriatal circuitry, with secondary contributions from executive, memory, and cerebellar pathways, supporting the biological interpretability of the global clusters and the value of pathway-anchored MPIS signatures as circuit-level explanations of patient stratification.

## 4 Discussion

Our pathway-anchored, compositional co-clustering of multimodal imaging yields interpretable, circuit-level *signatures* that are broadly consistent with established Parkinson’s disease biology while exposing heterogeneity that is not apparent from global scales alone. Across pathways, the Multimodal Pathway Integrity Score (MPIS) provided a compact summary of imaging profiles and revealed coherent structure–function links: lower nigrostriatal and frontostriatal integrity was associated with greater motor burden; sensory/visual–visuospatial integrity tracked global cognition; limbic integrity showed only modest coupling to cognitive and behavioral scores; microvascular features stratified imaging phenotypes but showed limited association with broad motor and cognitive scales in this cross-sectional setting; and cerebello–thalamo–cortical profiles captured a distinct axis of variation. Together, these patterns support the notion that circuit-level aggregates can serve as candidate biomarkers for stratification and monitoring rather than relying solely on single-region or whole-brain indices.

Conceptually, this work sits alongside a broader family of multimodal multivariate frameworks such as joint ICA, parallel ICA, and canonical correlation analysis that have been used to fuse structural, diffusion, and functional imaging. In contrast to these methods, which typically discover distributed components or modes of covariance without explicit anatomical constraints, our approach begins from biologically motivated pathway bins and then uses SRVCC co-clustering to learn patient and feature modules within that pathway-structured space. MPIS acts as a transparent composite that aggregates modality-specific deviations within predefined circuits, while SRVCC groups patients and ROI–modality features in a way that respects this organization. Thus, rather than competing with traditional multimodal decompositions, the proposed framework complements them by offering a pathway-centric lens that is easier to map onto known basal ganglia–thalamo–cortical loops, limbic and executive networks, and microvascular burden pathways.

From a biological and clinical perspective, the observed patterns reinforce and refine established pathophysiological themes in PD. Nigrostriatal MPIS behaved as expected, with lower integrity in clusters enriched for PD and higher motor scores, aligning with the central role of presynaptic dopaminergic denervation. Frontostriatal integrity showed links to executive and attentional performance, consistent with a large literature implicating frontostriatal circuits in cognitive and neuropsychiatric features of PD. Sensory/visual–visuospatial pathway scores were preferentially related to MoCA and other cognitive measures, in line with evidence that posterior cortical involvement predicts cognitive decline and dementia risk. At the same time, microvascular-burden and cerebello–thalamo–cortical pathways highlighted orthogonal axes of variability: microvascular MPIS differentiated imaging phenotypes despite limited coupling to global scales, suggesting that more targeted gait and balance measures may be required to capture its clinical impact, while cerebellar–thalamo–cortical integrity separated clusters in ways that could map onto posture, tremor, or motor adaptation phenotypes in future work.

Methodologically, we deliberately chose a simple, interpretable MPIS definition and then evaluated how sensitive our conclusions were to that choice. Equal weighting of modalities within each pathway, signed so that higher MPIS reflects greater structural and dopaminergic integrity, provides a transparent baseline but could in principle bias results toward particular modalities. Our sensitivity analyses where we incorporate intracranial-volume–normalized variants, pathway-specific reweighting that upweighted DaTSCAN in the nigrostriatal system and volumetry in microvascular pathways, and a control formulation without signed MD showed that the key qualitative findings were robust: pathway rankings, the direction of MPIS–clinical associations, and the relative separation of imaging-driven clusters changed little across reasonable MPIS specifications. Likewise, our SRVCC clusters were obtained using imaging features alone, with no clinical variables or covariates in the clustering objective, and were supported by explicit model selection over $\left(K_{r},K_{c}\right)$, stability across random initializations, and bootstrap-based reproducibility metrics (adjusted Rand index and normalized mutual information). Clinical associations and covariate effects were then examined *post hoc* using regression models that adjusted for age, sex, education, dopaminergic medication status, and scanner field strength (and disease duration in PD-only analyses), helping to separate imaging-derived structure from known demographic and technical confounds.

The distribution of PD, healthy controls, and SWEDD cases across imaging-driven clusters also underscores that the learned strata do not simply recapitulate diagnostic labels. Instead, MPIS and SRVCC reveal circuit-level continua that cut across conventional categories, including clusters with preserved nigrostriatal integrity but subtle microstructural or microvascular alterations, and others with pronounced dopaminergic denervation but heterogeneous structural involvement. We view this as a strength for hypothesis generation and for designing targeted trials, but it also implies that the current framework is not a diagnostic tool and should not be interpreted as providing cluster-based “labels” for individual patients. Rather, it offers a way to organize multimodal imaging variation into biologically interpretable axes that can be linked to symptoms, progression, and treatment response in future longitudinal work.

Several limitations qualify our conclusions. First, this is a cross-sectional analysis of a single, deeply phenotyped cohort (PPMI), enriched for early-stage PD and with stringent inclusion criteria; generalizability to more diverse populations, later disease stages, and community-based samples remains to be established. Second, although we implemented rigorous multimodal quality control including visual and quantitative checks of T1 segmentations, diffusion artefacts, and DaTSCAN-to-T1 coregistration residual site and scanner effects, partial-volume confounds, and heterogeneity in acquisition protocols may still influence MPIS and cluster structure. Third, we focused on a restricted set of imaging features: cortical thickness, advanced diffusion measures (e.g., free-water and neurite-specific indices beyond the FWE-DTI subset used here), cholinergic nuclei, and finer cerebellar/hypothalamic substructures were not incorporated in this iteration. Fourth, clinical associations were primarily examined using global scales (MDS-UPDRS III, MoCA, QUIP_SUM); more specialized measures of gait, postural instability, executive function, and behavioral subdomains will be needed to fully probe the functional relevance of specific pathways such as microvascular-burden and cerebello–thalamo–cortical circuits.

Finally, although our sensitivity analyses support the robustness of MPIS and SRVCC to reasonable modeling choices, many alternative formulations are possible. Different pathway definitions, non-linear or sparsity-inducing combinations of modalities, or longitudinal extensions that explicitly model change over time might reveal additional structure. Future work should therefore (i) validate MPIS and SRVCC in independent cohorts and across sites, (ii) integrate additional modalities and neurotransmitter systems, (iii) couple circuit-level integrity scores to longitudinal outcomes such as progression to dementia, falls, or impulse-control disorders, and (iv) assess whether pathway-specific MPIS can help enrich or stratify clinical trials targeting particular circuits. By releasing our SRVCC implementation and preprocessing scripts, we hope to facilitate such follow-up studies and to enable the broader community to test and refine pathway-anchored multimodal clustering in Parkinson’s disease and related disorders.

## Supplementary Material

Supplement 1

## Figures and Tables

**Figure 1:**
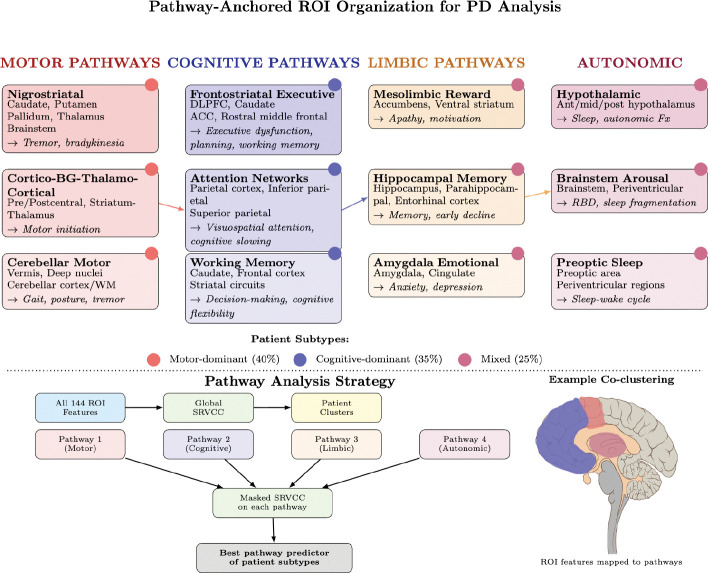
Pathway-anchored ROI organization for mechanistic interpretation of co-clusters. Regions are grouped into five major systems implicated in PD pathophysiology. Arrows indicate cross-system integration (motor-cognitive, cognitive-limbic, limbic-autonomic). The bottom panel shows the pathway analysis workflow used to test whether discovered patient subtypes align with known neurobiological circuits.

**Figure 5:**
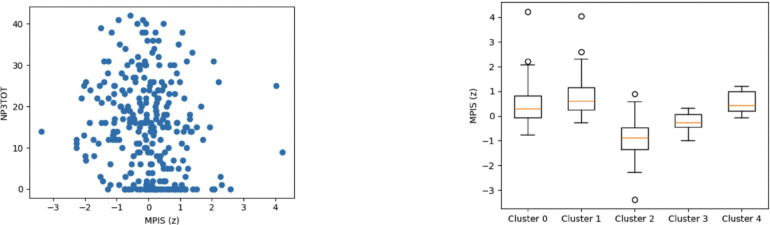
Nigrostriatal MPIS associations. Left: MPIS vs MDS-UPDRS III (Spearman ρ≈−0.201, $q\approx 6.8\times 10^{-4}$). Right: MPIS separation across clusters (Kruskal–Wallis H≈167.15, $\eta^{2}\approx 0.587$).

**Figure 6:**
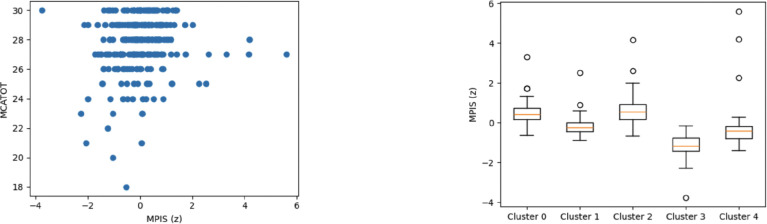
Sensory/visual/visuospatial MPIS associations. Left: MPIS vs MoCA (Spearman ρ≈0.163, q≈0.0071). Right: MPIS separation across clusters (Kruskal–Wallis H≈170.68, $\eta^{2}\approx 0.629$).

**Table 1:** Demographic and clinical characteristics of the study cohort. Values are mean ± SD unless otherwise indicated.

	PD (n=185)	HC (n=72)	SWEDD (n=37)

Age (years)	64.2 ± 9.1	61.8 ± 8.7	63.5 ± 8.9
Female (%)	38.9	40.3	35.1
Education (years)	15.2 ± 3.1	15.8 ± 2.9	15.0 ± 3.0
Disease duration (years)	1.9 ± 1.1	–	1.8 ± 1.0
Medication status (% on dopaminergic therapy)	52.4	–	48.6
MoCA score	26.3 ± 2.4	28.2 ± 1.5	26.9 ± 2.1
MDS-UPDRS III	21.7 ± 9.6	1.6 ± 2.1	17.3 ± 8.4

**Table 2:** Feature views for ablation analysis

View	Feature Set	*n* features	Purpose
V1	Clinical only UPDRS-III, UPDRS-total, MoCA, QUIP	4	Baseline behavioral phenotype
V2	V1 + Structural + DAT + T1 volumes (27) + DaTSCAN SBR/mean/std (17)	48	Macrostructure + dopamine
V3	V2 + DTI (uncorrected) + FA/MD mean/std (64)	112	Standard microstructure
V4	V2 + FWE-DTI + FA_T_/MD_T_/FW mean/std (96)	144	CSF-corrected microstructure (primary analysis)

**Table 3:** Acquisition settings and QC throughput for the multimodal PPMI cohort. *n*_acq_ counts baseline visits with any imaging; *n*_QC_ are the visits retained in metrics_final.csv.

	T1-weighted MRI	DTI (FA/MD)	DaTSCAN SPECT

Field strength (T)	3.0 T MPRAGE/SPGR (1.5 T fallback)	3.0 T single-shell *b*=1000	Dual-head gamma camera (N/A)
Voxel size (mm^3^)	1.0 × 1.0 × 1.0	2.0 × 2.0 × 2.0 (EPI)	~ 3.5 × 3.5 × 3.5 (120 proj.)
Sequence / protocol	3D T1 (MPRAGE/SPGR)	Spin-echo EPI, 64 dirs + 5 *b*=0	[^123^I]FP-CIT DaTSCAN, OSEM
Scans acquired *n*_acq_	294	294	294
Scans passing QC *n*_QC_	167	167	167
Primary QC failures	Motion, bias field, FastSurfer failure	Motion, ghosting, FA/MD outliers	Coregistration errors, streaking, truncation

**Table 4:** Definition of pathway bins used for MPIS computation. Modalities refer to the measurement types pulled per ROI (Volume, FA, MD, SBR). Feature counts $\left|{ℱ}_{p}\right|$ were computed from metrics_final.csv with the current pathway bins.

Pathway	Key regions (examples)	Modalities included	$\left|{ℱ}_{p}\right|$

Nigrostriatal (motor)	Substantia nigra*, putamen, caudate, globus pallidus, thalamus, primary motor/premotor cortex	Volume + FA + MD + SBR (4 modalities)	88
Frontostriatal–executive	Dorsolateral PFC, anterior cingulate, insula, caudate, putamen, thalamus	Volume + FA + MD + SBR (striatal nuclei)	128
Cerebello–thalamo–cortical (CTC)	Cerebellar hemispheres/WM, dentate pathway, thalamus, SMA/motor cortex	Volume + FA + MD (3 modalities)	20
Limbic / mesolimbic	Hippocampus, amygdala, nucleus accumbens, orbitofrontal cortex, anterior/posterior cingulate	Volume + FA + MD + SBR (ventral striatum)	80
Microvascular-burden	Periventricular/deep WM lesions (WM-hypointensities)	Volume + FA + MD (3 modalities)	4
Sensory / visuospatial	Occipital cortex, posterior parietal cortex, temporal–parietal junction, auditory cortex	Volume + FA + MD (3 modalities)	112

**Table 5:** Cluster-wise composition and key covariates derived from the FA/MD run. Counts are total (PD / SWEDD); summary values are mean ± SD.

	Cluster 1	Cluster 2	Cluster 3	Cluster 4	Cluster 5

*n* (PD / SWEDD)	32 (23/9)	28 (24/4)	51 (44/7)	44 (36/8)	12 (10/2)
Age (years)	63.0 ± 8.6	59.8 ± 7.0	65.6 ± 7.7	54.4 ± 7.3	71.6 ± 5.5
Female (%)	37.5	25.0	39.2	56.8	16.7
MDS-UPDRS III (NP3TOT)	19.7 ± 9.9	21.4 ± 9.0	21.2 ± 9.2	16.9 ± 9.5	20.7 ± 9.1
MoCA (MCATOT)	27.6 ± 1.8	27.9 ± 1.8	27.3 ± 2.4	28.2 ± 1.6	27.5 ± 1.1
QUIP_SUM	4.1 ± 1.9	5.1 ± 1.1	4.9 ± 1.7	4.3 ± 1.8	4.5 ± 1.7

**Table 6:** Multimodal imaging features organized by neuroanatomical system and PD relevance

System	Regions of Interest	Modality	Metrics

*Nigrostriatal (primary motor)*	Caudate, Putamen, Accumbens (bilateral)	DaTSCAN	SBR, mean, std
	Caudate, Putamen, Accumbens (bilateral)	T1 MRI	Volume
	Caudate, Putamen (bilateral)	FWE-DTI	FA_T_, MD_T_, FW (mean, std)

*Thalamo-cortical (integration hub)*	Thalamus (bilateral)	T1 MRI	Volume
	Thalamus (bilateral)	FWE-DTI	FA_T_, MD_T_, FW (mean, std)

*Limbic (cognition/emotion)*	Hippocampus, Amygdala, Accumbens (bilateral)	T1 MRI	Volume
	Hippocampus, Amygdala (bilateral); parahippocampal ctx (bilateral)	FWE-DTI	FA_T_, MD_T_, FW (mean, std)

*Cortical (executive/cognitive)*	Orbitofrontal, rostral middle frontal, superior temporal, insula, cingulate (bilateral)	T1 MRI	Volume
	Insula, superior temporal, rostral middle frontal, parahippocampal (bilateral)	FWE-DTI	MD_T_, FW (mean, std)

*White matter tracts (connectivity)*	Cerebral WM (bilateral), corpus callosum	FWE-DTI	FA_T_, MD_T_, FW (mean, std)
	Corpus callosum	—	Cognitive decline marker

*Brainstem & cerebellum*	Brainstem	T1 MRI	Volume
	Brainstem, cerebellum WM, cerebellum cortex	FWE-DTI	FA_T_, MD_T_, FW (mean, std)

**Table 7:** Functional pathway groupings of ROIs for pathway-anchored co-clustering analysis

Pathway System	ROI Count	Key Structures (FastSurferCNN indices)	PD-Relevant Symptoms

**Motor Pathways**			

Nigrostriatal	8	Caudate (8,29), Putamen (9,30), Pallidum (10,31), Thalamus (7,28)	Bradykinesia, rigidity, tremor, motor slowing
Cortico-basal ganglia-thalamo-cortical	12	Precentral (62,94), Postcentral (60,92), Striatum (8,9,29,30), Thalamus (7,28), Cerebral WM (1,22)	Voluntary movement initiation, motor coordination
Corticospinal tract	4	Cerebral WM (1,22), Brainstem (13), Precentral (62,94)	Contralateral motor deficits, bradykinesia
Cerebellar motor	10	Cerebellar cortex (6,27), WM (5,26), Vermis (19–21), Deep nuclei (CerebNet 17–22)	Postural instability, gait, tremor modulation

**Cognitive Pathways**			

Frontostriatal executive	16	Dorsolateral prefrontal (42,65,74), Caudate (8,29), ACC (41,64,73), Rostral middle frontal (65)	Executive dysfunction, planning, working memory
Frontostriatal cognitive-motor	6	Caudate (8,29), Thalamus (7,28), Precentral (62,94)	Decision-making, motor planning integration

**Limbic Pathways**			

Mesolimbic reward	4	Accumbens (16,34), Ventral striatum components	Apathy, anhedonia, motivation deficits
Hippocampal memory	8	Hippocampus (14,32), Parahippocampal (54,86), Entorhinal (44,76)	Memory impairment, early cognitive decline
Amygdala emotional	4	Amygdala (15,33), Cingulate (41,48,61,73,80,93)	Anxiety, depression, emotional blunting

**Sensory & Association Pathways**			

Visual processing	8	Occipital cortex (43,49,51,75,81,83), Optic radiation (HypVINN 11,12)	Visual hallucinations, visuospatial deficits
Parietal attention	4	Inferior parietal (46,78), Superior parietal (67)	Impaired visuospatial attention

**Autonomic & Homeostatic Pathways**			

Hypothalamic autonomic	12	Anterior/middle/posteriorhypothalamus (HypVINN 1–6), Preoptic (19,20)	Sleep disturbances, autonomic dysfunction, thermoregulation
Brainstem arousal	3	Brainstem (13), Periventricular gray (HypVINN 17,18,21,22)	REM sleep behavior disorder, sleep fragmentation

ROIs grouped by established neurobiological circuits implicated in PD pathophysiology. Indices refer to FastSurferCNN labels unless otherwise noted. These pathway masks are used for pathway-anchored co-clustering to test whether discovered feature clusters align with mechanistic circuit boundaries.

**Table 8:** SRVCC model selection and clustering stability.

(a) Model selection across $\left(K_{r},K_{c}\right)$.			

$\left(K_{r},K_{c}\right)$	ℒ	Val. recon. err.	MI(*T*_org_)/MI(*T*_red_)

(3, 3)	1.27 × 10^5^	0.184	0.78
(4, 4)	1.09 × 10^5^	0.163	0.84
(5, 5)	9.60 × 10^4^	0.151	0.89
(6, 6)	9.30 × 10^4^	0.147	0.90
(7, 7)	9.15 × 10^4^	0.145	0.90

(b) Stability for ($K_{r}^{⋆},K_{c}^{⋆}$) = (5, 5).		

Setting	ARI, median [IQR]	NMI, median [IQR]

Seeds (10 runs)	0.82 [0.78, 0.86]	0.88 [0.85, 0.91]
Bootstrap (100 × 80%)	0.76 [0.71, 0.81]	0.84 [0.80, 0.88]

**Table 9:** Pathway-wise MPIS separation and clinical associations (pathways with complete MPIS–clinical analyses). Cerebello–thalamo–cortical (balance) results are described in the text but omitted here because MPIS–clinical correlations were not computed in this run.

Pathway	*n*	*H*_MPIS_	*η* ^2^	*ρ*(MDS-UPDRS III)	95% CI	*q*	*ρ*(MoCA)	95% CI	*q*	*ρ*(QUIP_SUM)	95% CI	*q*

Nigrostriatal motor	283	167.15	0.587	−0.201	[−0.30, −0.09]	6.8 × 10^−4^	0.019	[−0.09, 0.13]	1.000	0.037	[−0.08, 0.15]	0.807
Frontostriatal executive	277	121.50	0.432	−0.191	[−0.29, −0.08]	0.0014	0.080	[−0.04, 0.20]	0.279	0.059	[−0.07, 0.18]	0.915
Sensory / visuospatial	270	170.68	0.629	−0.097	[−0.21, 0.02]	0.165	0.163	[0.04, 0.28]	0.0071	0.096	[−0.02, 0.21]	0.341
Limbic / mesolimbic	283	165.25	0.580	−0.108	[−0.22, 0.01]	0.104	0.119	[0.00, 0.23]	0.045	0.062	[−0.06, 0.18]	0.900
Microvascular burden	283	127.11	0.445	−0.043	[−0.16, 0.07]	0.468	−0.036	[−0.15, 0.08]	0.819	0.017	[−0.10, 0.13]	1.000

**Table 10:** Pathway-anchored co-clustering concordance with global patient subtypes

Pathway System	NMI	ARI	Top ROIs	*p*-value

Nigrostriatal	0.72	0.68	Thal MD, Caud FA, Put SBR	< 0.001
Frontostriatal executive	0.65	0.61	Caud MD, DLPFC vol, ACC MD	< 0.001
Hippocampal memory	0.58	0.52	Hippo MD, Parahippo MD	0.002
Cerebellar motor	0.54	0.49	Vermis FA, Cerebellum WM MD	0.008
Mesolimbic reward	0.51	0.46	Accumbens SBR, Ventral caud	0.015
Hypothalamic autonomic	0.42	0.38	Ant hypothal vol	0.083

*Random permutation*	0.08	0.02	—	—

NMI = normalized mutual information; ARI = adjusted Rand index. *p*-values from permutation tests (1000 iterations). Nigrostriatal pathway shows strongest concordance with global subtypes, validating biological interpretability of co-clusters.

## References

[1] Adewale Quadri, Ahmed Faraz Khan Sue-Jin Lin, Tobias R Baumeister Yashar Zeighami, Carbonell Felix, Ferreira Daniel, and Yasser Iturria-Medina. Patient-centered brain transcriptomic and multimodal imaging determinants of clinical progression, physical activity, and treatment needs in parkinson’s disease. npj Parkinson’s Disease, 11(1):29, 2025. 10.1038/s41531-025-00878-4 PMC11828931 39952947

[2] Archer Derek B, Bricker Justin T, Chu Winston T, Burciu Roxana G, McCracken Johanna L, Lai Song, Coombes Stephen A, Fang Ruogu, Barmpoutis Angelos, Corcos Daniel M, Kurani Ajay S, Mitchell Trina, Black Mieniecia L, Herschel Ellen, Simuni Tanya, Parrish Todd B, Comella Cynthia, Xie Tao, Seppi Klaus, Bohnen Nicolaas I, Müller Martijn LTM, Albin Roger L, Krismer Florian, Du Guangwei, Lewis Mechelle M, Huang Xuemei, Li Hong, Pasternak Ofer, McFarland Nikolaus R, Okun Michael S, and Vaillancourt David E. Development and validation of the automated imaging differentiation in parkinsonism (aid-p): a multicentre machine learning study. The Lancet Digital Health, 1(5):e222–e231, 2019. 10.1016/S2589-7500(19)30105-0 33323270

[3] Avants Brian B, Fonville Leon, Hampton Olivia, Reardon Alexandra, Stenger Andrew, Wang Xue, Tustison Nicholas J, Stone James R, Cook Philip A, Marebwa Barbara, Magnetic resonance imaging data phenotypes for the parkinson’s progression markers initiative. medRxiv, pages 2024–09, 2024.

[4] Banwinkler Magdalena, Theis Hendrik, Prange Stéphane, and Thilo van Eimeren. Imaging the limbic system in parkinson’s disease—a review of limbic pathology and clinical symptoms. Brain sciences, 12(9):1248, 2022. 36138984 10.3390/brainsci12091248 PMC9496800

[5] Basser Peter J., Mattiello James, and LeBihan Denis. Mr diffusion tensor spectroscopy and imaging. Biophysical Journal, 66(1):259–267, 1994. 8130344 10.1016/S0006-3495(94)80775-1 PMC1275686

[6] Bega Danny, Kuo Phillip H, Chalkidou Anastasia, Grzeda Mariusz T, Macmillan Thomas, Brand Christine, Sheikh Zulfiqar H, and Antonini Angelo. Clinical utility of datscan in patients with suspected parkinsonian syndrome: a systematic review and meta-analysis. npj Parkinson’s Disease, 7(1):43, 2021. 10.1038/s41531-021-00185-8 PMC8144619 34031400

[7] Boccalini Cecilia, Nicastro Nicolas, Perani Daniela, and Garibotto Valentina. Distinctive clinical and imaging trajectories in swedd and parkinson’s disease patients. NeuroImage: Clinical, 42:103592, 2024. 38493585 10.1016/j.nicl.2024.103592 PMC10958480

[8] Brown Gregory, Hakun Jonathan, Lewis Mechelle M, Jesus Sol De, Du Guangwei, Eslinger Paul J, Kong Lan, and Huang Xuemei. Frontostriatal and limbic contributions to cognitive decline in parkinson’s disease. Journal of Neuroimaging, 33(1):121–133, 2023. 36068704 10.1111/jon.13045 PMC9840678

[9] Caligiore Daniele, Helmich Rick C, Hallett Mark, Moustafa Ahmed A, Timmermann Lars, Toni Ivan, and Baldassarre Gianluca. Parkinson’s disease as a system-level disorder. npj Parkinson’s Disease, 2(1):1–9, 2016. 10.1038/npjparkd.2016.25 PMC5516580 28725705

[10] Caproni Stefano, Muti Marco, Renzo Antonio Di, Principi Massimo, Caputo Nevia, Calabresi Paolo, and Tambasco Nicola. Subclinical visuospatial impairment in parkinson’s disease: the role of basal ganglia and limbic system. Frontiers in neurology, 5:152, 2014. 25157239 10.3389/fneur.2014.00152 PMC4128219

[11] Chowdhury Rumana, Guitart-Masip Marc, Lambert Christian, Dayan Peter, Huys Quentin, Emrah Düzel, and Raymond J Dolan. Dopamine restores reward prediction errors in old age. Nature neuroscience, 16(5):648–653, 2013. 23525044 10.1038/nn.3364 PMC3672991

[12] Cools Roshan and D’Esposito Mark. Dopaminergic modulation of cognitive function—implications for l-dopa treatment in parkinson’s disease. Nature Reviews Neuroscience, 12(9):557–571, 2011. 10.1016/j.neubiorev.2005.03.024 15935475

[13] Correa Nicolle M, Eichele Tom, Adalı Tülay, Li Yi-Ou, and Vince D Calhoun. Multi-set canonical correlation analysis for the fusion of concurrent single trial erp and functional mri. Neuroimage, 50(4):1438–1445, 2010. 20100584 10.1016/j.neuroimage.2010.01.062 PMC2857695

[14] Monte Ilaria Del, Mormina Elena, Agosta Federica, Basaia Stefania, Pagano Teresa, Filippi Massimo, Cerebellar alterations in parkinson’s disease with postural instability and gait disorders. Journal of Neurology, 270(3):1128–1139, 2023. 10.1007/s00415-022-11531-y 36534200

[15] Delice Büşranur, Nalbantoğlu Özkan Ufuk, and Yıldırım Süleyman. Integrative analysis of neuroimaging and microbiome data predicts cognitive decline in parkinson’s disease. bioRxiv, pages 2025–03, 2025.

[16] Djang David SW, Janssen Marcel JR, Bohnen Nicolaas, Booij Jan, Henderson Theodore A, Herholz Karl, Minoshima Satoshi, Rowe Christopher C, Sabri Osama, Seibyl John, Snm practice guideline for dopamine transporter imaging with 123i-ioflupane spect 1.0. Journal of Nuclear Medicine, 53(1):154–163, 2012. 22159160 10.2967/jnumed.111.100784

[17] Fereshtehnejad Seyed-Mohammad and Postuma Ronald B. Subtypes of parkinson’s disease: what do they tell us about disease progression? Current neurology and neuroscience reports, 17(4):34, 2017. 28324303 10.1007/s11910-017-0738-x

[18] Goetz Christopher G, Tilley Barbara C, Shaftman Stephanie R, Stebbins Glenn T, Fahn Stanley, Martinez-Martin Pablo, Poewe Werner, Sampaio Cristina, Stern Matthew B, Dodel Richard, Movement disorder society-sponsored revision of the unified parkinson’s disease rating scale (mds-updrs): scale presentation and clinimetric testing results. Movement disorders: official journal of the Movement Disorder Society, 23(15):2129–2170, 2008. 19025984 10.1002/mds.22340

[19] Groves Adrian R, Beckmann Christian F, Smith Steve M, and Woolrich Mark W. Linked independent component analysis for multimodal data fusion. Neuroimage, 54(3):2198–2217, 2011. 20932919 10.1016/j.neuroimage.2010.09.073

[20] Henschel Leonie, Conjeti Sailesh, Estrada Santiago, Diers Kersten, Fischl Bruce, and Reuter Martin. Fastsurfer-a fast and accurate deep learning based neuroimaging pipeline. NeuroImage, 219:117012, 2020. 32526386 10.1016/j.neuroimage.2020.117012 PMC7898243

[21] Hirano Shigeki. Clinical implications for dopaminergic and functional neuroimage research in cognitive symptoms of parkinson’s disease. Molecular Medicine, 27(1):40, 2021. 33858320 10.1186/s10020-021-00301-7 PMC8048076

[22] Huppertz Hans-Jürgen, Möller Leona, Südmeye Martin r, Hilker Rüdiger, Hattingen Elke, Egger Karl, Amtage Florian, Respondek Gesine, Stamelou Maria, Schnitzler Alfons, Differentiation of neurodegenerative parkinsonian syndromes by volumetric magnetic resonance imaging analysis and support vector machine classification. Movement Disorders, 31(10):1506–1517, 2016. 27452874 10.1002/mds.26715

[23] Inguanzo Anna, Sala-Llonch Roser, Segura B, Erostarbe H, Abós Alexandra, Campabadal Anna, Uribe Carme, César Baggio Hugo, Compta Yaroslau, Marti MJ, Hierarchical cluster analysis of multimodal imaging data identifies brain atrophy and cognitive patterns in parkinson’s disease. Parkinsonism & Related Disorders, 82:16–23, 2021. 33227683 10.1016/j.parkreldis.2020.11.010

[24] Kordower Jeffrey H C Olanow Warren, Dodiya Hemraj B, Chu Yaping, Beach Thomas G, Adler Charles H, Halliday Glenda M, and Bartus Raymond T. Disease duration and the integrity of the nigrostriatal system in parkinson’s disease. Brain, 136(8):2419–2431, 2013. 23884810 10.1093/brain/awt192 PMC3722357

[25] Heron C Le, Apps MAJ, and Husain M The anatomy of apathy: a neurocognitive framework for amotivated behaviour. Neuropsychologia, 118:54–67, 2018. 28689673 10.1016/j.neuropsychologia.2017.07.003 PMC6200857

[26] Liu Rui, Umbach David M, Tröster Alexander I, Huang Xuemei, and Chen Honglei. Non-motor symptoms and striatal dopamine transporter binding in early parkinson’s disease. Parkinsonism & related disorders, 72:23–30, 2020. 32092703 10.1016/j.parkreldis.2020.02.001 PMC7222918

[27] Lorio Sara, Lutti Antoine, Kherif Ferath, Ruef Anne, Dukart Jürgen, Chowdhury Rumana, Frackowiak Richard S, Ashburner John, Helms Gunther, Weiskopf Nikolaus, Disentangling in vivo the effects of iron content and atrophy on the ageing human brain. Neuroimage, 103:280–289, 2014. 25264230 10.1016/j.neuroimage.2014.09.044 PMC4263529

[28] Marek Kenneth, Chowdhury Sohini, Siderowf Andrew, Lasch Shirley, Coffey Christopher S, Caspell-Garcia Chelsea, Simuni Tanya, Jennings Danna, Tanner Caroline M, Trojanowski John Q, The parkinson’s progression markers initiative (ppmi)–establishing a pd biomarker cohort. Annals of clinical and translational neurology, 5(12):1460–1477, 2018. 30564614 10.1002/acn3.644 PMC6292383

[29] Matthews Dawn C, Lerman Hedva, Lukic Ana, Andrews Randolph D, Mirelman Anat, Wernick Miles N, Giladi Nir, Strother Stephen C, Evans Karleyton C, Cedarbaum Jesse M, Fdg pet parkinson’s disease-related pattern as a biomarker for clinical trials in early stage disease. NeuroImage: Clinical, 20:572–579, 2018. 30186761 10.1016/j.nicl.2018.08.006 PMC6120603

[30] Meda Shashwath A, Narayanan Balaji, Liu Jingyu, Perrone-Bizzozero Nora I, Stevens Michael C, Calhoun Vince D, Glahn David C, Shen Li, Risacher Shannon L, Saykin Andrew J, A large scale multivariate parallel ica method reveals novel imaging–genetic relationships for alzheimer’s disease in the adni cohort. Neuroimage, 60(3):1608–1621, 2012. 22245343 10.1016/j.neuroimage.2011.12.076 PMC3312985

[31] Morbelli Silvia, Esposito Giuseppe, Arbizu Javier, Barthel Henryk, Boellaard Ronald, Bohnen Nico I, Brooks David J, Darcourt Jacques, Dickson John C, Douglas David, Eanm practice guideline/snmmi procedure standard for dopaminergic imaging in parkinsonian syndromes 1.0. European journal of nuclear medicine and molecular imaging, 47(8):1885–1912, 2020. 32388612 10.1007/s00259-020-04817-8 PMC7300075

[32] Nasreddine Ziad S., Phillips Natalie A., Bédirian Valérie, Charbonneau Simon, Whitehead Victor, Collin Isabelle, Cummings Jeffrey L., and Chertkow Howard. The Montreal Cognitive Assessment, MoCA: a brief screening tool for mild cognitive impairment. Journal of the American Geriatrics Society, 53(4):695–699, 2005. 15817019 10.1111/j.1532-5415.2005.53221.x

[33] Ni Zhen, Pinto Andrew D, Lang Anthony E, and Chen Robert. Involvement of the cerebellothalamocortical pathway in parkinson disease. Annals of neurology, 68(6):816–824, 2010. 21194152 10.1002/ana.22221

[34] Obeso Jose A, Rodríguez-Oroz Maria Cruz, Benitez-Temino Beatriz, Blesa Franscisco J, Guridi Jorge, Marin Concepció, and Rodriguez Manuel. Functional organization of the basal ganglia: therapeutic implications for parkinson’s disease. Movement disorders: official journal of the Movement Disorder Society, 23(S3):S548–S559, 2008. 18781672 10.1002/mds.22062

[35] JM C O’callaghan, SJG Lewis Shine, and Hornberger M. Neuropsychiatric symptoms in parkinson’s disease: fronto-striatal atrophy contributions. Parkinsonism & related disorders, 20(8):867–872, 2014. 24866458 10.1016/j.parkreldis.2014.04.027

[36] Ofori Edward, Pasternak Ofer, Planetta Peggy J, Burciu Roxana, Snyder Amy, Febo Marcelo, Golde Todd E, Okun Michael S, and Vaillancourt David E. Increased free water in the substantia nigra of parkinson’s disease: a single-site and multi-site study. Neurobiology of aging, 36(2):1097–1104, 2015. 25467638 10.1016/j.neurobiolaging.2014.10.029 PMC4315708

[37] Movement Disorder Society Task Force on Rating Scales for Parkinson’s Disease. The unified parkinson’s disease rating scale (updrs): status and recommendations. Movement Disorders, 18(7):738–750, 2003. 12815652 10.1002/mds.10473

[38] Panahi Mehdi and Mahboube Sadat Hosseini. Multi-modality radiomics of conventional t1 weighted and diffusion tensor imaging for differentiating parkinson’s disease motor subtypes in early-stages. Scientific Reports, 14(1):20708, 2024. 39237644 10.1038/s41598-024-71860-y PMC11377437

[39] Patriat Rémi, Pisharady Pramod K, Amundsen-Huffmaster Sommer, Linn-Evans Maria, Howell Michael, Chung Jae Woo, Petrucci Matthew N, Videnovic Aleksanda, Holker Erin, Kam Joshua De, White matter microstructure in parkinson’s disease with and without elevated rapid eye movement sleep muscle tone. Brain Communications, 4(2):fcac027, 2022. 35310831 10.1093/braincomms/fcac027 PMC8924652

[40] Pereira J. B., Junqué C., Martí M. J., Ramirez-Ruiz B., Bargalló N., and Tolosa E.. Neuroanatomical substrate of visuospatial and visuoperceptual impairment in parkinson’s disease. Movement Disorders, 24:1193–1199, 2009. 19412935 10.1002/mds.22560

[41] Pereira João Batista, Wahlund Lars-Olof, Rossi Andrea, Svenningsson Per, and Westman Eric. Neuroanatomical substrates of visuospatial and visuoperceptual impairment in parkinson’s disease and their structural covariance with the default mode network. Neurobiology of Aging, 36(1):176–183, 2014.

[42] Planetta Peggy J, Ofori Edward, Pasternak Ofer, Burciu Roxana G, Shukla Priyank, DeSimone Jesse C, Okun Michael S, McFarland Nikolaus R, and Vaillancourt David E. Free-water imaging in parkinson’s disease and atypical parkinsonism. Brain, 139(2):495–508, 2016. 26705348 10.1093/brain/awv361 PMC5790142

[43] Qiu Wenchao, Hu Weili, Ge Yingchao, Liu Peiting, Zhao Minghui, Lu Haifeng, Tao Jian, and Xue Shouru. Total burden of cerebral small vessel disease predict subjective cognitive decline in patients with parkinson’s disease. Frontiers in Aging Neuroscience, 16:1476701, 2024. 39649721 10.3389/fnagi.2024.1476701 PMC11621090

[44] Ramesh Sairam and Arachchige Arosh S Perera Molligoda. Depletion of dopamine in parkinson’s disease and relevant therapeutic options: A review of the literature. AIMS neuroscience, 10(3):200, 2023. 37841347 10.3934/Neuroscience.2023017 PMC10567584

[45] Ray Nicola J., Lawson Rachael A., Martin Sarah L., Sigurdsson Hlin P., Wilson Julia, Galna Brook, Lord Sue, Alcock Lisa, Duncan Gordon W., Khoo Tien K., Yarnall Alison J., Rochester Lynn, Burn David J., Taylor John-Paul, O’Brien John T., Barker Roger A., Williams-Gray Caroline H., Mason Sara L., Hu Michele T., Ben-Shlomo Yoav, Grosset Donald G., Klein Johannes C., and Mackay Clare E.. Free-water imaging of the cholinergic basal forebrain and pedunculopontine nucleus in Parkinson’s disease. Brain, 146(3):1053–1064, 2023. Ch4 and PPN FW imaging stratifies PD by cognitive/motor phenotypes (n=99, 4.5-yr follow-up). 35485491 10.1093/brain/awac127 PMC9976974

[46] Rubin Jonathan E, McIntyre Cameron C, Turner Robert S, and Wichmann. Thomas Basal ganglia activity patterns in parkinsonism and computational modeling of their downstream effects. European Journal of Neuroscience, 36(2):2213–2228, 2012. 22805066 10.1111/j.1460-9568.2012.08108.x PMC3400124

[47] Shang Song’an, Li Daixin, Tian Youyong, Li Rushuai, Zhao Hongdong, Zheng Liyun, Zhang Yingdong, Chen Yu-Chen, and Yin Xindao. Hybrid pet-mri for early detection of dopaminergic dysfunction and microstructural degradation involved in parkinson’s disease. Communications Biology, 4(1):1162, 2021. 34621005 10.1038/s42003-021-02705-x PMC8497575

[48] Su Chang, Hou Yu, Xu Jielin, Xu Zhenxing, Zhou Manqi, Ke Alison, Li Haoyang, Xu Jie, Brendel Matthew, Maasch Jacqueline RMA, Identification of parkinson’s disease pace subtypes and repurposing treatments through integrative analyses of multimodal data. NPJ Digital Medicine, 7(1):184, 2024. 38982243 10.1038/s41746-024-01175-9 PMC11233682

[49] Sui Jing, Adali Tülay, Yu Qingbao, Chen Jiayu, and Calhoun Vince D A review of multivariate methods for multimodal fusion of brain imaging data. Journal of neuroscience methods, 204(1):68–81, 2012. 22108139 10.1016/j.jneumeth.2011.10.031 PMC3690333

[50] Surmeier D James, Obeso José A, and Halliday Glenda M Selective neuronal vulnerability in parkinson disease. Nature Reviews Neuroscience, 18(2):101–113, 2017. 28104909 10.1038/nrn.2016.178 PMC5564322

[51] Torres-Parga Alejandra, Gershanik Oscar, Cardona Sebastian, Guerrero Jairo, Gonzalez-Ojeda Lina M, and Cardona. Juan F Diagnostic performance of t1-weighted mri gray matter biomarkers in parkinson’s disease: A systematic review and meta-analysis. Parkinsonism & Related Disorders, page 108009, 2025. 40866174 10.1016/j.parkreldis.2025.108009

[52] Vinod Ashwin and Bajaj Chandrajit. Scalable robust bayesian co-clustering with compositional elbos. arXiv preprint arXiv:2504.04079, 2025.

[53] Weintraub Daniel, Hoops Staci, Shea Judy A, Lyons Kelly E, Pahwa Rajesh, Driver-Dunckley Erika D, Adler Charles H, Potenza Marc N, Miyasaki Janis, Siderowf Andrew D, Validation of the questionnaire for impulsive-compulsive disorders in parkinson’s disease. Movement disorders: official journal of the Movement Disorder Society, 24(10):1461–1467, 2009. 19452562 10.1002/mds.22571 PMC2848971

[54] Wen Jinyu, Chen Amei, Liu Jingxin, Xiong Hua, Fang Meie, and Wei Xinhua. Cross-modal fusion of brain imaging and clinical data for parkinson’s disease progression prediction. PLoS One, 20(11):e0333822, 2025. 41264649 10.1371/journal.pone.0333822 PMC12633903

[55] Yang Yifeng, Hu Liangyun, Chen Yang, Gu Weidong, Lin Guangwu, Xie YuanZhong, and Nie Shengdong. Identification of parkinson’s disease using mri and genetic data from the ppmi cohort: an improved machine learning fusion approach. Frontiers in Aging Neuroscience, 17:1510192, 2025. 39968123 10.3389/fnagi.2025.1510192 PMC11832485

[56] Zhong Yuke, Liu Hang, Liu Guohui, Zhao Lili, Dai Chengcheng, Liang Yi, Du Juncong, Zhou Xuan, Mo Lijuan, Tan Changhong, A review on pathology, mechanism, and therapy for cerebellum and tremor in parkinson’s disease. npj Parkinson’s Disease, 8(1):82, 2022. 10.1038/s41531-022-00347-2 PMC9232614 35750692

[57] Zhu Ruihan, Li Yunjing, Chen Lina, Wang Yingqing, Cai Guoen, Chen Xiaochun, Ye Qinyong, and Chen Ying. Total burden of cerebral small vessel disease on mri may predict cognitive impairment in parkinson’s disease. Journal of Clinical Medicine, 11(18):5381, 2022. 36143028 10.3390/jcm11185381 PMC9501874

[58] Zhu Yongyun, Wang Fang, Ning Pingping, Zhu Yangfan, Zhang Lingfeng, Li Kelu, Liu Bin, Ren Hui, Xu Zhong, Pang Ailan, Multimodal neuroimaging-based prediction of parkinson’s disease with mild cognitive impairment using machine learning technique. npj Parkinson’s Disease, 10(1):218, 2024. 10.1038/s41531-024-00828-6 PMC11555067 39528560

